# A Power Conversion Technique with Hierarchical Equalization Charging Topology for LiFePO_4_ Batteries

**DOI:** 10.3390/mi12091014

**Published:** 2021-08-26

**Authors:** Kuo-Ching Tseng, Hao-Shiang Huang, Chun-An Cheng

**Affiliations:** 1 Department of Electronic Engineering, National Kaohsiung University of Science and Technology, Kaohsiung City 82445, Taiwan; jerry@nkust.edu.tw (K.-C.T.); u0452811@nkust.edu.tw (H.-S.H.); 2 Department of Electrical Engineering, I-Shou University, Kaohsiung City 84001, Taiwan

**Keywords:** battery, converter, equalizer

## Abstract

An energy-storage scheme with hierarchical equalization charging topology applied in a series-connected battery system is proposed in this paper. The proposed hierarchical equalization charging topology (HECT), which combines an equalizer-within module (EWM) and an equalizer between the modules (EBM), is able to rapidly achieve charging balance among a large number of cells in battery modules. The EWM is composed of a buck–boost converter, while a flyback converter constitutes the EBM. Besides, the voltage of each cell in battery modules can be accurately monitored by utilizing the proposed HECT control architecture. In addition, fewer circuit elements are required in the proposed battery equalization system and a faster balancing speed can be achieved. Satisfactory experimental results were obtained by using 12 LiFePO_4_ batteries, and the performance was improved by about 50% in reducing the battery voltage deviation realized in the proposed battery balancing system, which verified the function of the proposed HECT scheme.

## 1. Introduction

**Figure 1:**
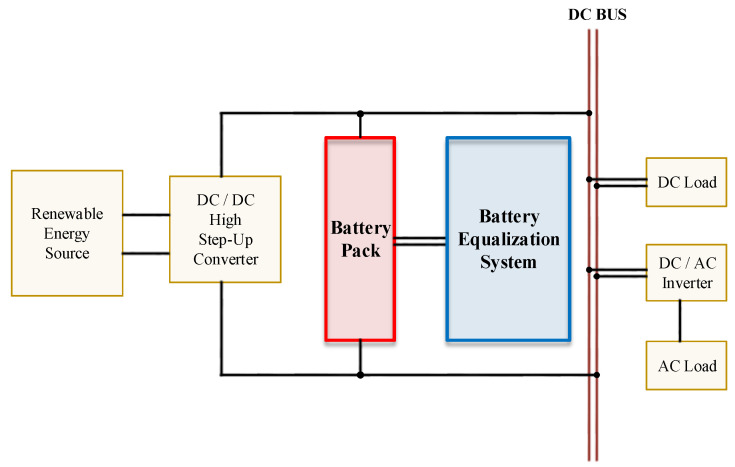
The architecture of a renewable energy system.

Energy conservation and reduction of carbon dioxide to avoid the greenhouse effect are the goals of clean and green energy promoted by countries all over the world today. Batteries are an important part of clean and green energy technology and are consistent with this goal. The architecture of a renewable energy system is shown in Figure 1. The battery pack not only supplies power to the DC load through the DC bus, but also supplies power to the AC load through the inverter. Due to the high conversion efficiency of batteries, which is far better than the internal combustion engine of gasoline vehicles (GV) [1], the electric vehicle (EV) industry has developed rapidly and has become a hot topic in recent years. On the other hand, portable and wearable storage products are popular development trends for electronic products. Therefore, batteries are also necessary and indispensable components in mobile devices and wearable devices, and their importance is self-evident. The voltage level of the battery is not sufficient to power portable and wearable storage products; for example, the voltage inside a single cell of a Li-ion battery, a lead–acid battery, and a Ni–MH battery is 3.3 V~3.7 V, 2.4 V~2.8 V, and 1.2 V, respectively [2,3,4,5,6]. This means that batteries need to be connected in series to effectively power portable and wearable storage products. For instance, there are 7104 Li-ion cells in a Tesla Model S EV, and these supply 400 V to the motor [7].

Each individual battery has slightly different characteristics, in terms of manufacturing tolerances or usage conditions, such as inherent resistance, capacity, and life cycle. The unbalance of the series-connected battery will be very serious after many times of charging and discharging [8]. When the battery is in undercharged or overcharged condition, it can cause battery breakdown or even a thermal runaway. Therefore, when the battery is charging or discharging, a battery balancing system is needed to average all of the voltages of the battery in series connection to ensure its safety [9]. To ensure safety and optimize performance, all batteries need to be monitored by an electronic battery management system (BMS) [10,11]. Many equalizing methods for batteries have been proposed [12,13,14]. In terms of hardware, battery equalizers can be classified as dissipative (passive) and non-dissipative (active) types.

The dissipation equalizer [15,16,17] consumes the charge of the overcharged battery through a resistor until the battery voltage reaches equilibrium. To reduce power losses, the shunt resistors can be replaced by Zener diodes or connected with active switches. Dissipative equalizers are suitable in terms of low capacitance and low power rating, but are not suitable for large battery packs.

Dealing with the voltage difference of the battery cells through the dissipative battery equalizer will result in significant power loss on the resistor. Saving energy and reducing unnecessary energy consumption is an increasingly important issue. Therefore, the non-dissipative battery equalizer is regarded as the main research topic in recent years [18,19,20,21,22,23,24,25,26,27,28,29,30,31].

In response to the above requirements, this paper proposes an energy storage scheme with a hierarchical equalization charging topology, which is applied to a series-connected battery system. In addition, in the proposed method, charge balance among a large number of batteries can be quickly achieved, and a faster balance speed can be achieved.

## 2. The Proposed Hierarchical Equalization Charging Topology

**Figure 2:**
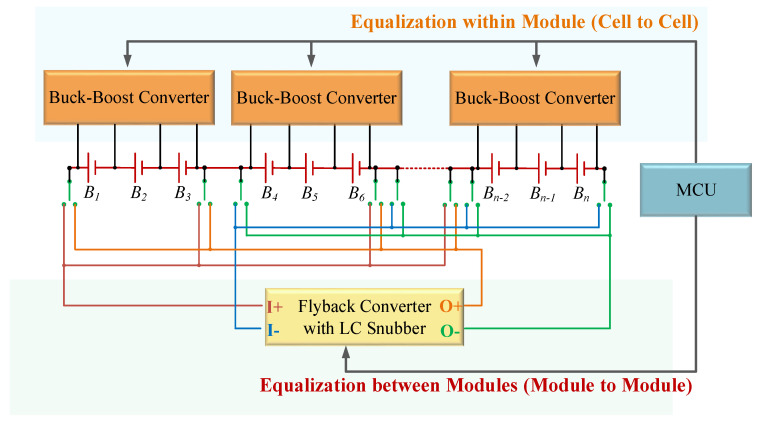
The proposed battery equalizer system with hierarchical equalization charging topology.

The proposed battery equalizer system with hierarchical equalization charging topology (HECT) is shown in Figure 2. This system is comprised of the equalizer-within module (EWM), the equalizer between the modules (EBM), and the master control unit (MCU). The main components of the proposed battery equalizer system are introduced as follows.

1. Equalizer-Within Module (EWM)

The cell-by-cell topology is applied in the equalizer-within module. Each battery module is composed of three cells. The main architecture of this equalizer consists of the concentration stage and redistribution stage. Thus, it has great performance and works efficiently with a simple control process.

2. Equalizer between the Modules (EBM)

Each battery pack, where three cells in it have already been equalized, is considered as a single module. Since energy can be transferred between two modules via a long-distance charging or discharging path, the module-to-module topology is the best choice for this equalizer system. The flyback converter with an LC snubber is used to extract energy from the high-voltage module and send it to the low-voltage module. Module selection is operated by relays on four transmission buses (including blue, green, orange, and brown buses).

3. Master Control Unit (MCU)

The master control unit is responsible for voltage sensing, data processing, and driving the equalizers. The MCU of the proposed equalizer system includes LTC-6802-2, arduino UNO, arduino MEGA, and FPGA.

The advantages of the proposed battery equalizer system with hierarchical equalization charging topology are listed as follows.

(1) The most advantageous feature of the cell-by-cell topology is its modularity. In addition, the proposed system applies this topology to each module of three cells. Therefore, it is easy to manufacture when used in a large number of battery packs. In addition, the energy exchange between only three cells in a single module has the advantage of reducing the loss caused by the transmission path.
(2) Since each group of three battery cells is considered as a single module, the number of floating switches required between the modules of the isolated power converter structure can be greatly reduced.
(3) Whether it is an equalizer within a module or an equalizer between modules, the converter used in the equalization system can greatly accelerate the equalization speed.

### 2.1. Equalizer-Within Module (EWM)

**Figure 3:**
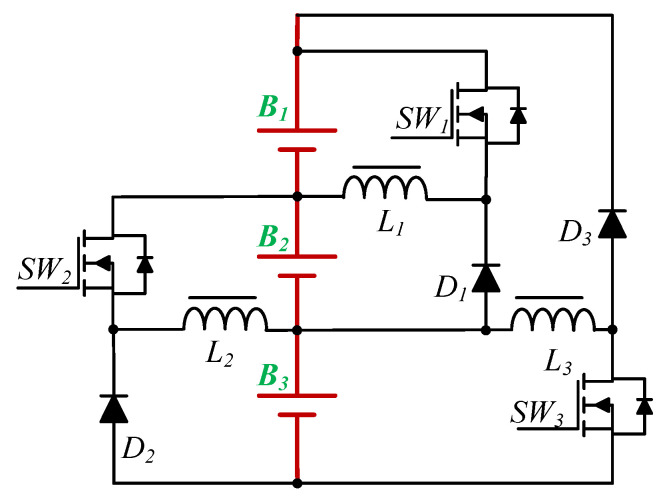
The proposed EWM.

**Figure 4:**
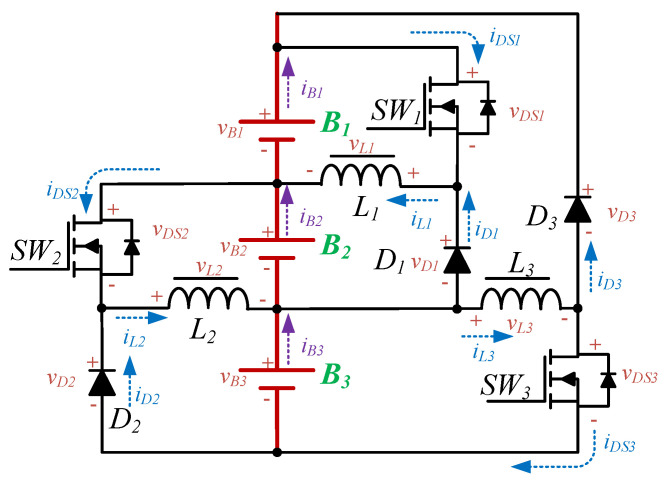
The definition of the component polarity in the EWM.

The proposed EWM is shown in Figure 3. This equalizer is a unidirectional buck–boost converter, which is composed of a concentration stage and a redistribution stage; the operation principles for each stage are described in the next section. The definition of the component polarity of the equalizer is shown in Figure 4. The energy is collected and redistributed by the battery at the bottom of the module. In addition, in order to achieve high-speed equalization, the main control unit (MCU) will determine the on/off state, and will adjust the duty cycle of the respective gate-driving signals of all power switches according to the battery voltage.

#### 2.1.1. Operation Principle of the EWM

**Figure 5:**
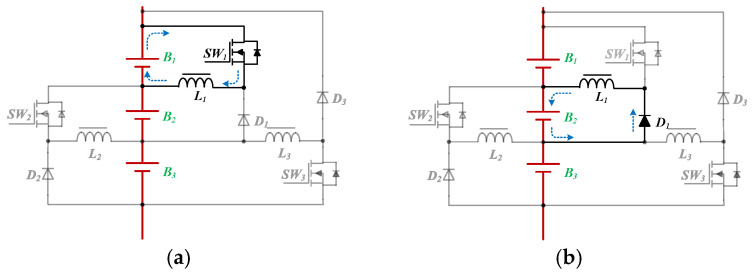
The current path of the concentration stage in Mode 1: (**a**) The switch *SW_1_* is turned on (**b**) The switch *SW_1_* is turned off.

The current path of the concentration stage in Mode 1 is shown in Figure 5. In Figure 5a, switch *SW*_1_ is turned on, and inductor *L*_1_ is charged by the battery *B*_1_. In Figure 5b, switch *SW*_1_ is turned off, and the inductor *L*_1_ provides energy to the battery *B*_2_. Thus, the energy is unidirectional shifted from *B*_1_ to *B*_2_.

**Figure 6:**
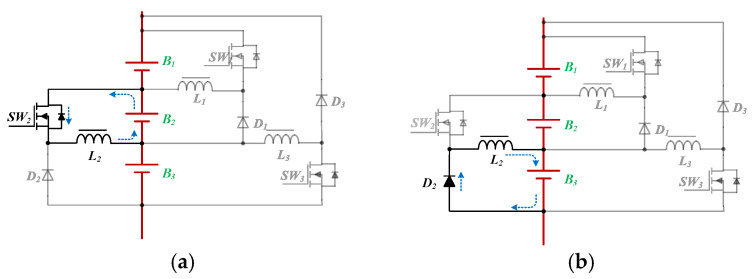
The current path of the concentration stage in Mode 2: (**a**) The switch *SW_2_* is turned on (**b**) The switch *SW_2_* is turned off.

The current path of the concentration stage in Mode 2 is shown in Figure 6. Similar to Mode 1, in Mode 2, the energy transferring between batteries *B*_2_ and *B*_3_ is shown in Figure 6a,b, respectively.

**Figure 7:**
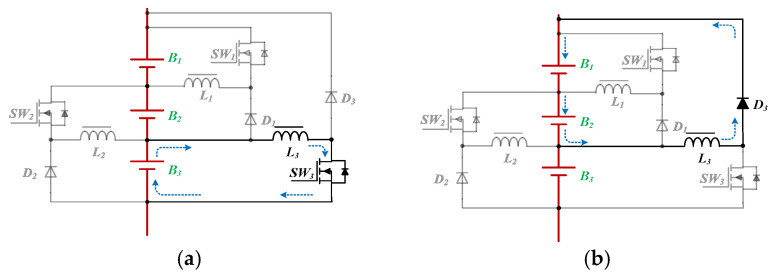
The current path of the redistribution stage in Mode 3: (**a**) The switch *SW_3_* is turned on (**b**) The switch *SW_3_* is turned off.

The current path of the redistribution stage in Mode 3 is shown in Figure 7. In Figure 7a, the power switch *SW*_3_ is turned on, and the battery *B*_3_ provides energy to the inductor *L*_3_ through *SW*_3_. In Figure 7b, the switch *SW*_3_ is turned off, and the inductor *L*_3_ supplies energy to batteries *B*_1_ and *B*_2_.

#### 2.1.2. Design of the Energy Storage Elements (Inductors) in the EWM

In the proposed EWM, digital control is adopted, and the duty cycle of the power switch is adjusted to achieve the balance of the battery. In addition, the inductor inside the equalizer is designed to operate in discontinuous conduction mode (DCM) to control the battery to achieve equilibrium. The inductance *L_n_* in the EWM operated at boundary-conduction-mode (BCM) can be derived as [32]:

(1) $$L_{n}\leq \frac{{\left(V_{Bn}\cdot D\right)}^{2}}{2\cdot f\cdot P_{o}}$$

where *V_Bn_* is the voltage of the battery; *D* is the duty ratio of the power switch; *f* is the switching frequency; *P_o_* is the output power.

### 2.2. Equalizer between Modules (EBM)

**Figure 8:**
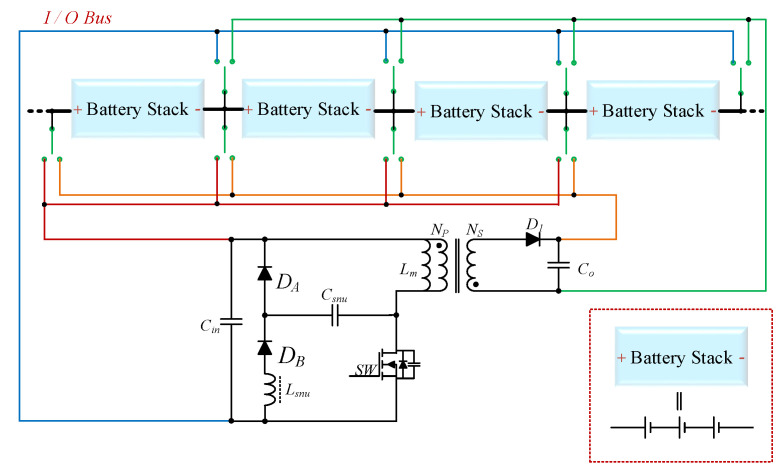
The proposed equalizer between modules.

The proposed equalizer between the modules (EBM) is depicted in Figure 8. The EBM uses a traditional flyback converter because it is suitable for low and medium power ratings. In order to reduce losses and improve conversion efficiency, the energy in the leakage inductance can be recycled by a non-dissipative snubber on the primary side of the transformer. Referring to [33], for clamping the voltage to alleviate the voltage stress of the main switch *SW*, the value of the snubber capacitor *C_snu_* is given by:

(2) $$C_{snu}=\frac{L_{lk}\cdot \left(i_{Llk\left(MAX\right)}-i_{Llk\left(min\right)}\right)}{v_{Csnu\left(MAX\right)}+v_{Csnu\left(min\right)}}$$

In this equation, the *i_Llk(MAX)_* is the maximum current of leakage inductor during the switch-on period. Moreover, the *i_Llk(min)_* is the maximum current of leakage inductor during the switch-on period. The *v_Csnu(MAX)_* is the maximum snubber capacitor voltage. On the contrary, the *v_Csnu(min)_* is the minimum snubber capacitor voltage.

In addition, the snubber is capable to recycle the energy stored in the leakage inductor *L_lk_* of the transformer. The snubber inductor *L_snu_* can be expressed as [33]:

(3) $$L_{snu}=\frac{D_{min}{}^{2}}{C_{snu}\cdot {\left(\pi \cdot f\right)}^{2}}$$

In this equation, the *D_min_* is the minimum duty cycle of operating equalizer. The *f* is the operating frequency.

### 2.3. Control Architecture of the Proposed Equalizer System

A previous reference has proved that the control program is the most important factor affecting the speed of battery balancing [33]. In addition, the control program can adjust the battery balance state according to various conditions of the battery pack.

**Figure 9:**
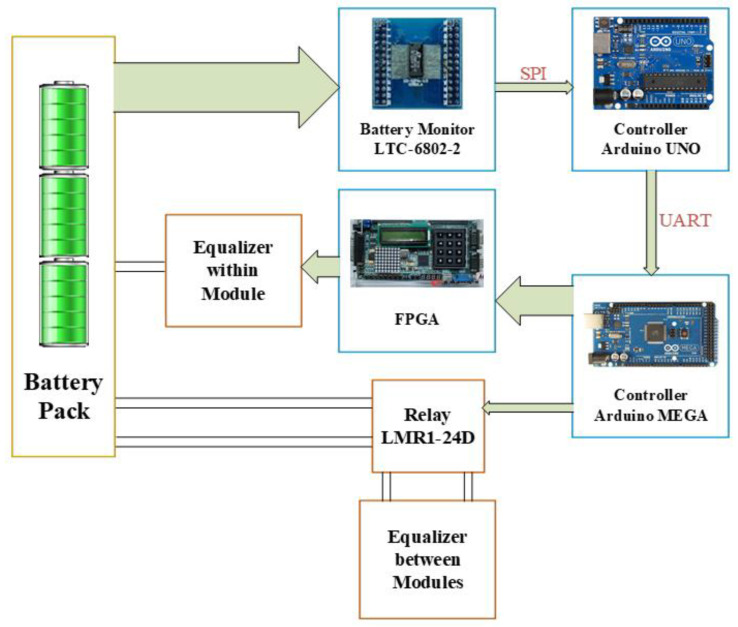
The control architecture of the proposed equalizer system.

The control architecture of the proposed equalizer system is shown in Figure 9. The controller includes the battery monitor LTC-6802-2, center micro controller unit Arduino UNO and multiplexed controller Arduino MEGA and FPGA. In addition, the battery monitor is responsible for showing the real-time status of cell equalization.

**Figure 10:**
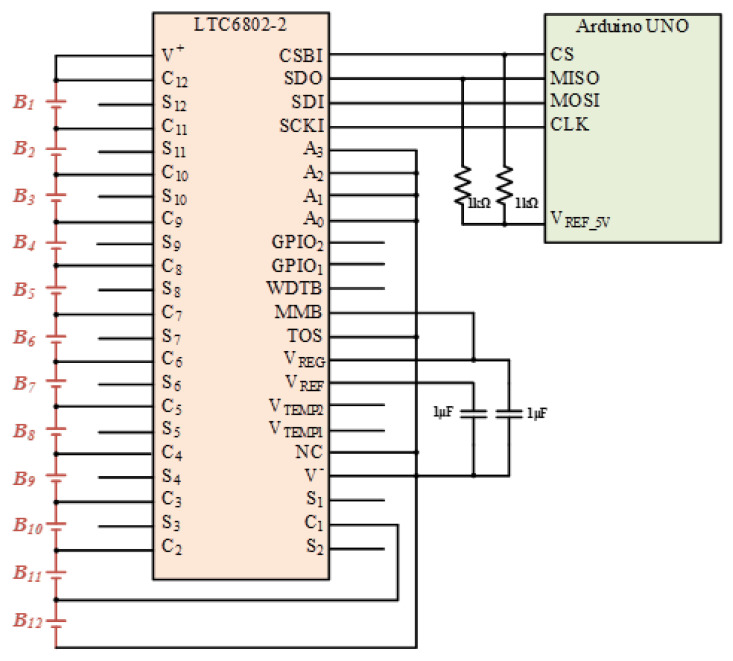
The circuit diagram of LTC-6802-2 connected with the Arduino UNO.

**Figure 11:**
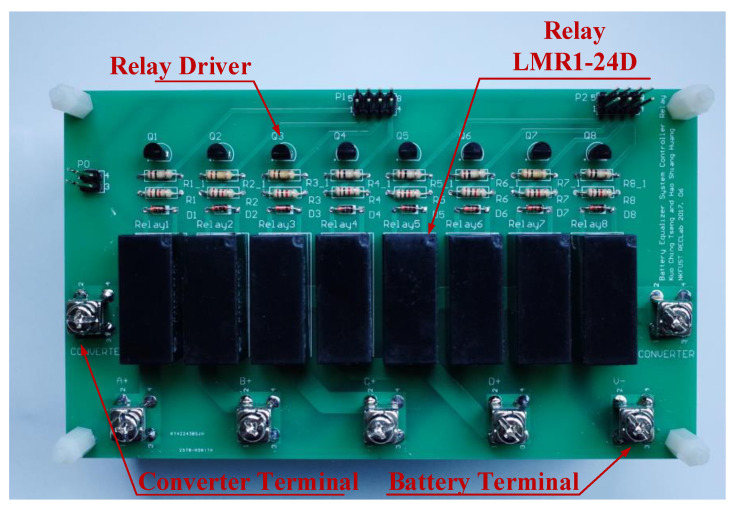
The photo of the utilized relay LMR1-24D in the proposed battery equalization system.

**Figure 12:**
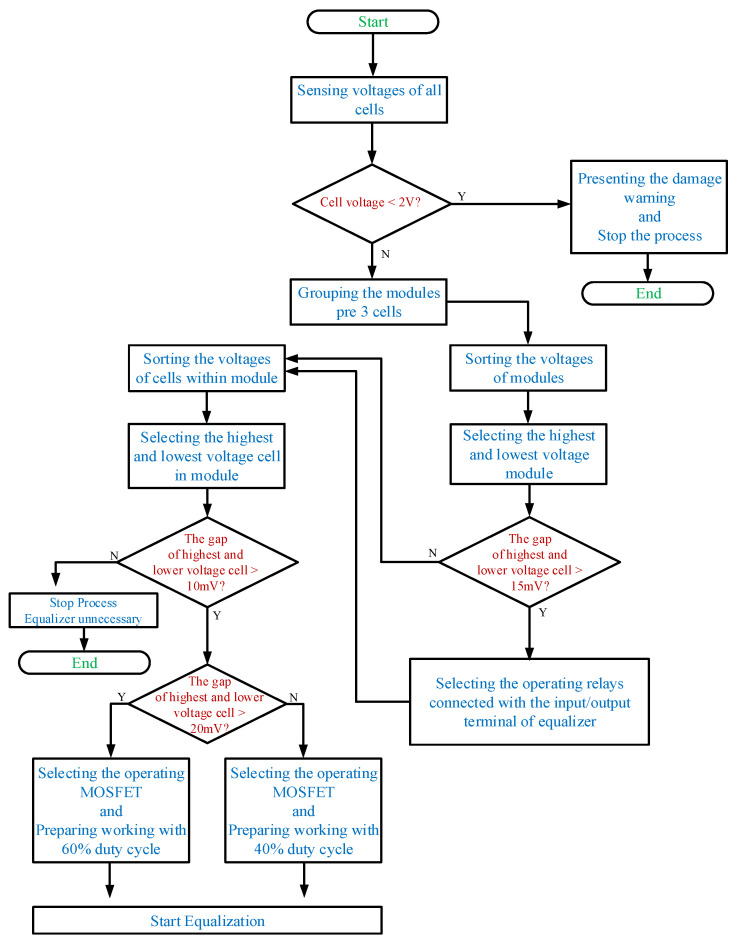
The flow chart of the control program for EWM.

**Figure 13:**
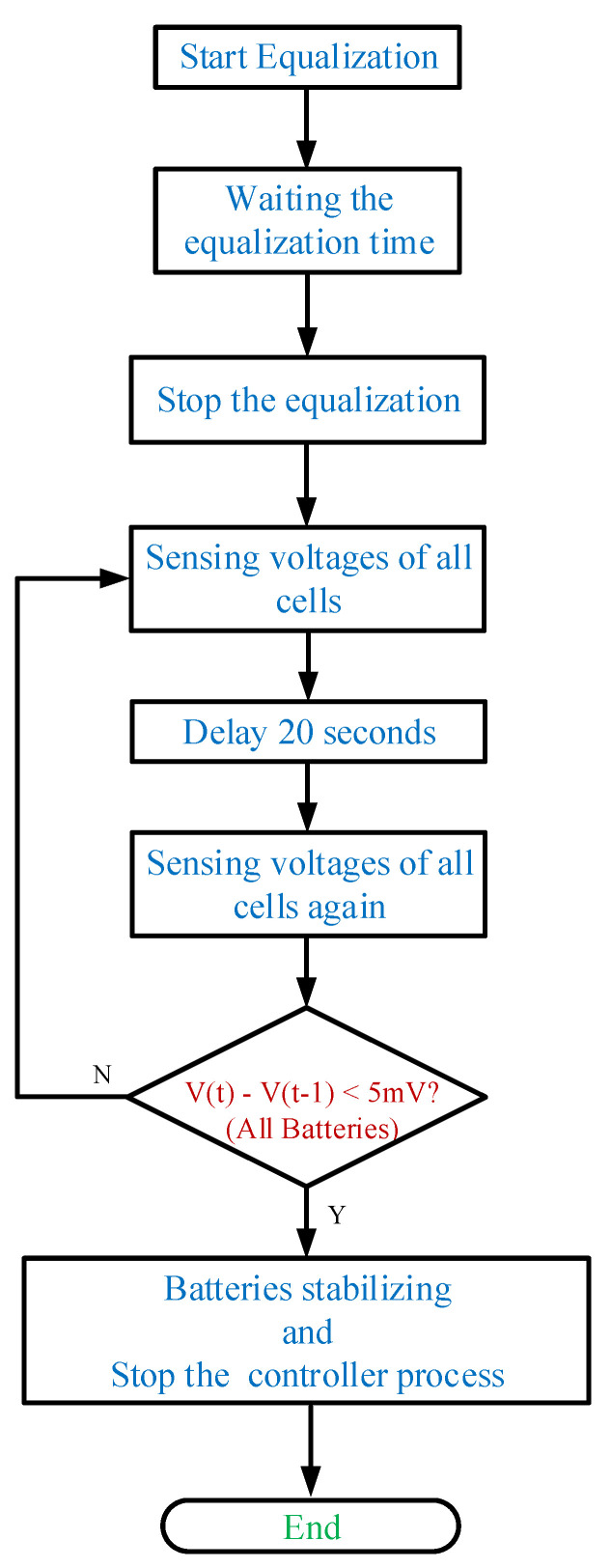
The flow chart of the control program for EBM.

For a large number of cells, the controller system is divided into three parts. The battery voltage data is collected by the battery monitor LTC-6802-2, and then transmitted to the central controller Arduino UNO through the SPI interface. The circuit diagram of LTC-6802-2 connected with the Arduino UNO is shown in Figure 10. In addition, the battery data will be displayed on the monitor at the same time, and then sent from Arduino UNO to the Arduino MEGA through the UART interface. Arduino MEGA arranges and specifies the battery to be balanced, and then Arduino MEGA and FPGA send gate-driving signals to each power switch for controlling the equalizer within module. Meanwhile, the Arduino MEGA sends commands to the Relays (LMR1-24D) for controlling the equalizer between module. In addition, the photo of the utilized relays (LMR1-24D) along with the relay drivers in the proposed battery equalization system is shown in Figure 11. The flow charts of the control program for EWM and EBM are shown in Figure 12 and Figure 13, respectively.

## 3. Experimental Results of Proposed Equalization System

For power batteries in electric vehicles, LiFePO_4_ batteries are considered to be the fastest growing in the lithium-ion battery family, because LiFePO_4_ batteries have many advantages. In terms of safety, LiFePO_4_ batteries do not have safety hazards such as overheating and explosion. In terms of cost, the cathode material of LiFePO_4_ batteries is cheaper than LiNiO_2_ batteries and LiCoO_2_ batteries. In addition, the cycle life of LiFePO_4_ batteries is four to five times that of lithium batteries, and the weight is reduced by 30 to 50%. Therefore, the LiFePO_4_ battery, which is PC40100LEP_10Ah, is employed in the experiments of the proposed battery equalization system. In addition, the nominal capacity and voltage of the utilized LiFePO_4_ battery are 10 Ah and 3.3 V, respectively.

### 3.1. The Experimental Results of Equalizer within Module

**Figure 14:**
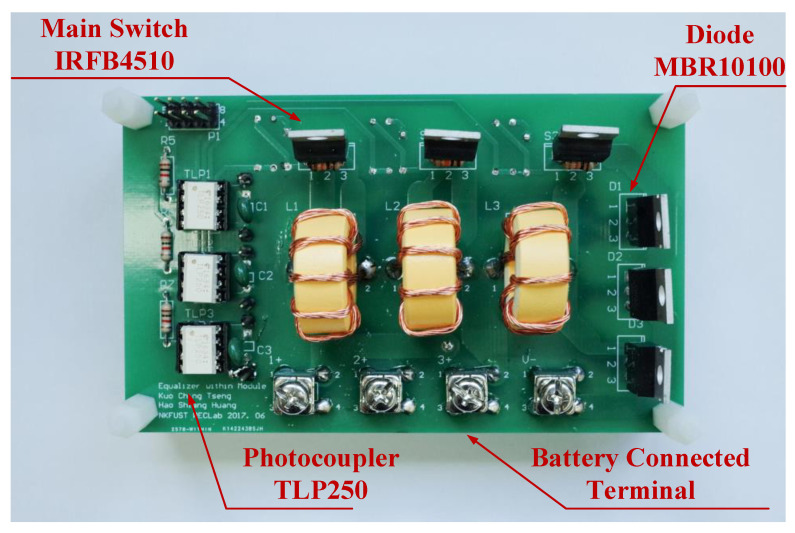
The circuit photo of the proposed equalizer within module.

**Figure 15:**
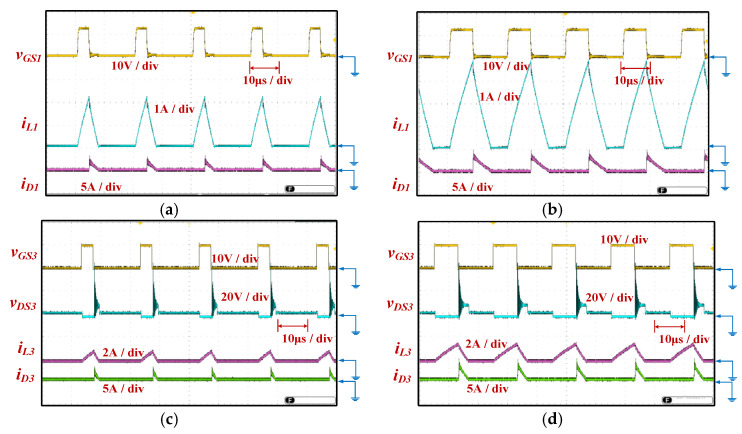
The experimental waveforms of the proposed equalizer within module: Measured *v_GS1_*, *i_L1_*, and *i_D1_* at (**a**) 20% and (**b**) 40% duty cycle of *SW_1_*; Measured *v_GS3_*, *v_DS3_*, *i_L3_*, and *i_D3_* at (**c**) 20% and (**d**) 40% duty cycle of *SW_3_*.

The parameters and components utilized in the prototype circuit of the proposed equalizer within module are presented in Table 1. The circuit photo of the proposed equalizer within module used for experiment is shown in Figure 14, and the experimental waveforms of the proposed equalizer within module are shown in Figure 15.

Figure 15a,b respectively show the gate-driving signal *v_GS_*_1_ with 20% and 40% duty cycle of the power switch *SW_1_*, the current *i_L_*_1_ of the inductor *L*_1_, and the current *i_D_*_1_ of the diode *D*_1_.

Figure 15c,d respectively show the gate-driving signal *v_GS_*_3_ with duty cycles 20% and 40%, the voltage *v_DS_*_3_ of the power switch *SW*_3_, the current *i_L_*_3_ of inductor *L*_3_, and the current *i_D_*_3_ of diode *D*_3_. In addition, the controller used in the proposed system can adjust the balancing speed of the battery cells by changing the duty cycle of the power switch.

### 3.2. The Experimental Results of Equalizer between Modules

The parameters and components utilized in the prototype circuit of the proposed equalizer between modules are shown in Table 2.

**Figure 16:**
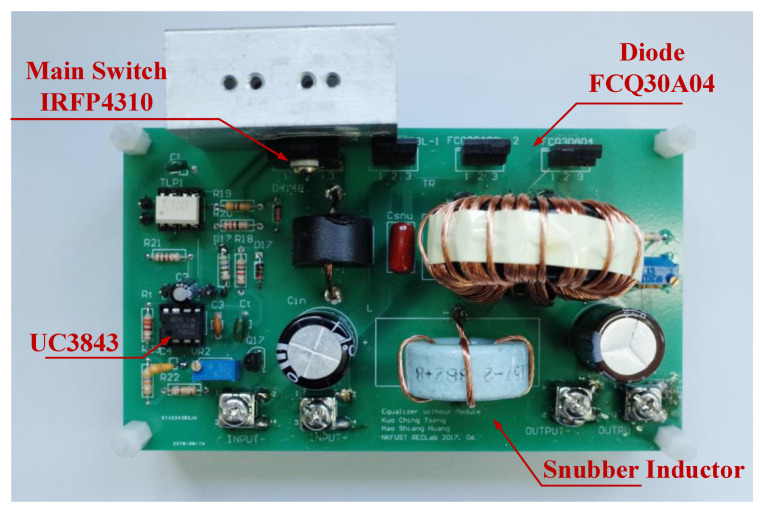
The circuit photo of the proposed equalizer between modules.

**Figure 17:**
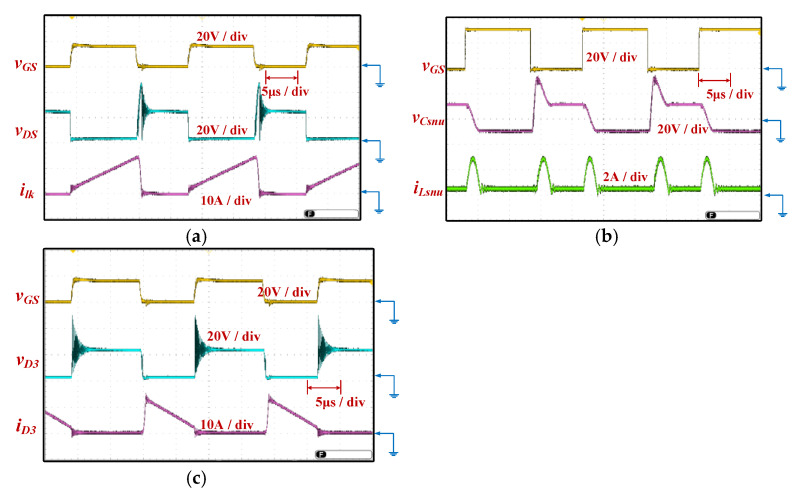
The experimental waveforms of the proposed equalizer between modules: (**a**) Measured *v_GS_*, *v_DS_*, and *i_lk_* (**b**) Measured *v_GS_*, *v_csnu_*, and *i_Lsnu_* (**c**) Measured *v_GS_*, *v_D3_*, and *i_D3_*.

The circuit photo of the proposed equalizer between modules used for experiment is shown in Figure 16. The experimental waveforms of proposed equalizer at the full load are shown in Figure 17.

Figure 17a exhibits the gate-driving signal *v_GS_* and voltage *v_DS_* of the main switch SW, and the leakage inductor current *i_lk_*. Figure 17b shows the gate-driving signal of switch SW, the voltage *v_Csnu_* of the snubber capacitor *C_snu_*, and the current *i_Lsnu_* of the snubber inductor *L_snu_*. Although there is a spike voltage caused by leakage inductor and parasitic capacitance C*_ds_* of SW, the energy can be recovered to input and output via the snubber circuit. The experimental waveforms of diode *D*_3_ are depicted in Figure 17c, which include diode voltage *v_D_*_3_ and current *i_D_*_3_ along with the gate-driving signal *v_GS_*.

### 3.3. The Experimental Results of the Proposed Battery Equalization System

**Figure 18:**
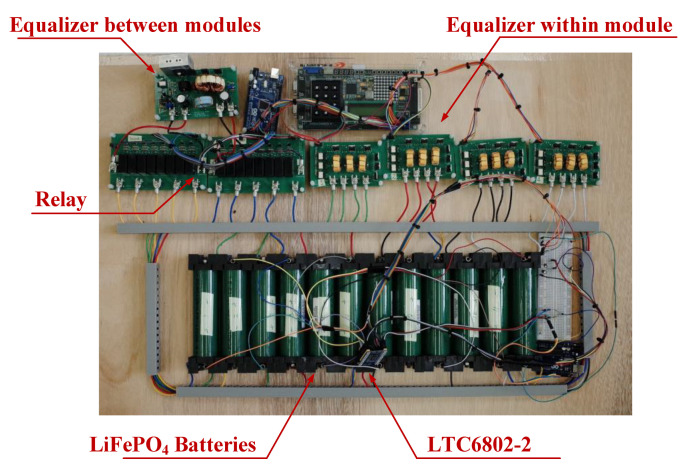
The circuit photo of the overall proposed battery equalization system.

Twelve LiFePO_4_ batteries with uneven battery voltage are applied to the proposed battery equalization system with hierarchical equalization charging topology to demonstrate the function, and the circuit photo of the overall proposed battery equalization system is shown in Figure 18.

**Figure 19:**
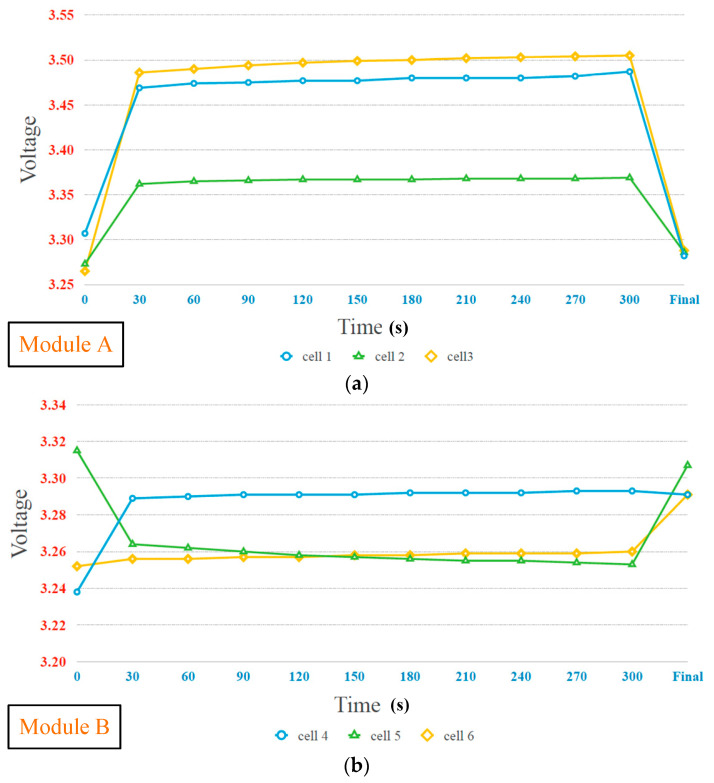

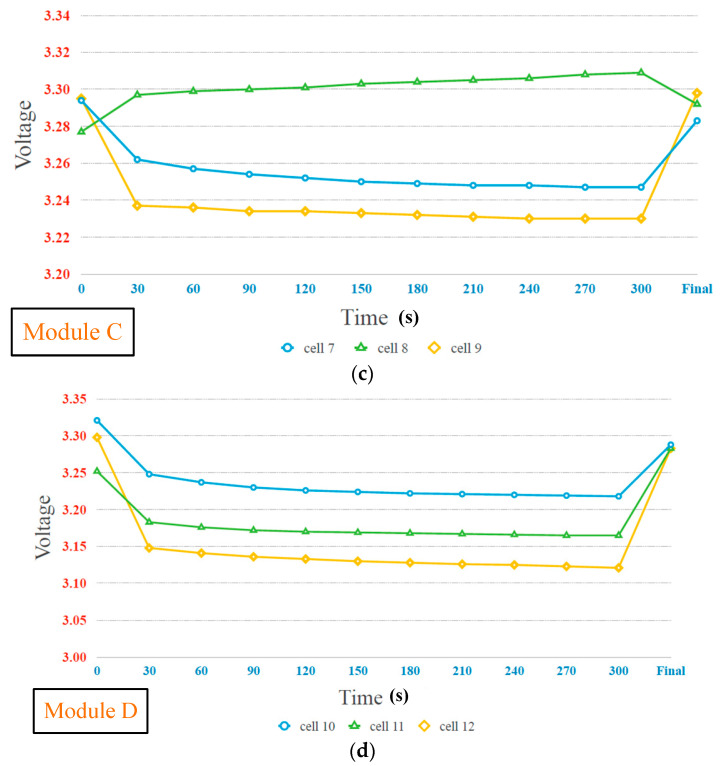
The experiment results of measured voltages in each modules at static equalization process: (**a**) Module A (**b**) Module B (**c**) Module C (**d**) Module D.

**Figure 20:**
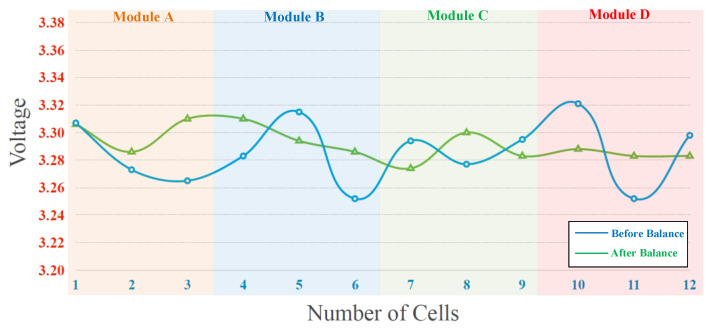
The overall experiment result of static equalization in the proposed battery equalization system.

The experimental results of the proposed battery equalization system are presented in two parts: static equalization and dynamic equalization. The experimental battery pack consisted of four modules. The detailed static equalization process and measured voltage of each battery cell in Module A, Module B, Module C, and Module D are shown in Figure 19a–d, respectively. In addition, the voltages of each cells in the Modules are measured per 30 s, and the total battery balancing time is 5 min. The battery voltage varies greatly from 0 s to 30 s and 300 s to final, because the ohmic polarization voltage of the battery responds quickly with the change of the battery charging and discharging state; the concentration polarization voltage response is slow and irregular. Therefore, during the charge/discharge phase, there is a significant difference in the polarization voltage of the battery. On the other hand, the overall experimental result of static equalization in the proposed battery equalization system is presented in Figure 20. As shown in Figure 20, the initial voltage of each cell before battery equalization is indicated by the blue line. In addition, the final voltage of each cell after battery equalization is indicated by a green line. Obviously, the difference in battery voltage is gradually eliminated by the proposed battery equalization scheme. Since the initial battery pack voltage of Module A is the lowest, and the initial battery pack voltage of Module D is the highest, the proposed battery equalization system performs the operation of equalizer between modules. Therefore, the battery pack voltage of Module A increases significantly, as shown in Figure 19a. Moreover, the equalizer within modules is effective, which can prevent the over-discharge of the No. 11 battery cell in Module D, as shown in Figure 19d. In addition, the maximum voltage difference of the total battery cells before and after balancing is reduced from 70 to 36 mV, as shown in Figure 18.

On the other hand, the balance of the battery in the process of charging and discharging, that is, dynamic equalization, is both complicated and necessary. The dynamic balance experiment of the battery pack includes the charging and discharging process, and the charging and discharging currents are both 0.2 C. The total operating time of the dynamic balance experiment is 5 min.

**Figure 21:**
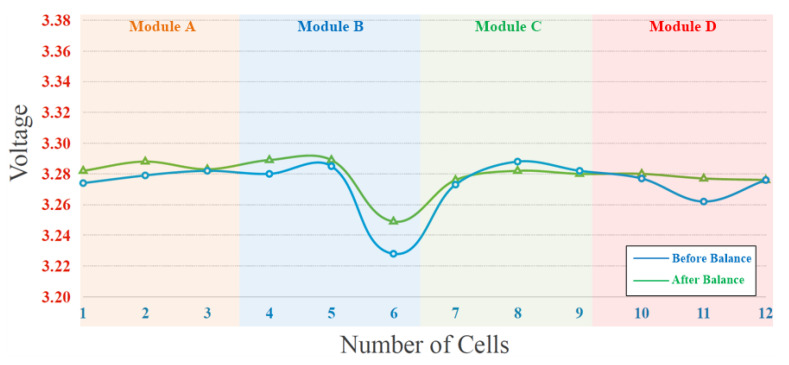
The experiment result of dynamic equalization during charging.

The experimental result of dynamic equalization of the battery pack in the charging process is shown in Figure 21. During the charging process of the battery pack, the voltage difference between Module C and Module D is greatly improved. Since the voltage of the No. 6 battery cell is significantly lower than other batteries, the charging intensity is increased by the proposed equalizer. In addition, the maximum voltage difference of the total battery cells before and after balancing is reduced from 60 to 40 mV, as shown in Figure 21.

**Figure 22:**
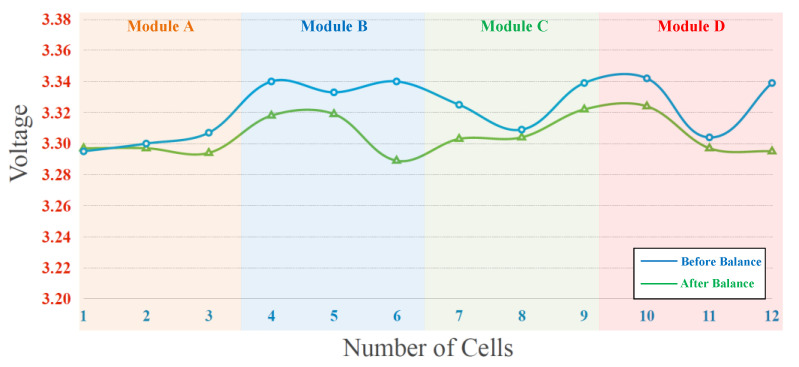
The experiment result of dynamic equalization during discharging.

Besides, the experiment result of dynamic equalization of the battery pack in the discharging process is presented in Figure 22. Since the battery cell voltage of module A is the lowest among the four modules, the proposed equalizer activates dynamic equalization to achieve battery voltage balance. In addition, the maximum voltage difference of the total battery cells before and after balancing is reduced from 47 to 35 mV, as shown in Figure 22.

## 4. Discussion

From experimental results shown in Figure 19a–d, it can be calculated that the average voltage of twelve cells within four modules is 3.2823 V before battery equalization. The maximum and minimum deviations with respect to the average voltage before battery equalization are 38.7 and 30.3 mV, respectively. Besides, it can be calculated that the average voltage of twelve cells within four modules is 3.2893 V after battery equalization. The maximum and minimum deviations with respect to the average voltage after battery equalization are 17.7 and 7.3 mV, respectively. In addition, the maximum deviations with respect to the average voltage before and after battery equalization are 1.18% and 0.538%, respectively. Therefore, in the proposed battery equalization system, an improvement of about 50% can be achieved in reducing the deviation of the battery voltage.

Table 3 shows comparisons of performance between the existing balance methods and the proposed HECT one. The switched capacitor method has high balance efficiency, and the multi-winding transformer method has a fast balance speed. Besides, the methods of bleed resister, analog shunting, and switched capacitor have excellent balance control. In addition, the advantages of the proposed HECT scheme are good balance efficiency, good balance speed, moderate balance control, and it is suitable for modular design.

## 5. Conclusions

This paper proposes a power conversion technique with a hierarchical equalization charging topology, which is suitable for series-connected battery systems. The scheme combines an equalizer within a module and an equalizer between modules. The proposed battery equalizer improves the unbalanced condition of the battery voltage, thereby ensuring the safety of the battery and prolonging the service life of the battery. The proposed equalizer reduces the steps required for battery balancing through modular batteries and a hierarchical architecture, so as to accelerate the speed of battery balancing. During the experiment, 12 LiFePO_4_ batteries were used. The experimental results show that the battery voltage deviation is reduced by about 50%, which proves the function and performance of the proposed battery equalizer with a hierarchical equalizing charging topology.

## Figures and Tables

**Table 1:** The parameters and components utilized in the prototype circuit of the proposed equalizer within module.

Parameter/Component	Value
Input Voltage *V_i_*	2.1 V~3.65 V
Output Voltage *V_o_*	2.1 V~3.65 V
Switching Frequency	50 kHz
Maximum Power *P*_o_	15 W
Inductors *L*_1_*, L*_2_*, L*_3_	6 μH
Power Switches *SW*_1_*, SW*_2_*, SW_3_*	IRFB4510
Rectifier Didoes *D*_1_*, D*_2_*, D_3_*	MBR10100
Photocoupler	TLP250

**Table 2:** The parameters and components utilized in the prototype circuit of the proposed equalizer between modules.

Parameter/Component	Value
Input Voltage *V_i_*	9.9 V
Output Voltage *V_o_*	10.95 V~12 V
Switching Frequency	50 kHz
Maximum Power *P*_o_	40 W
Duty Ratio	0.55
Turns Ratio (*N_P_*:*N_S_*)	1:1
Magnetizing Inductor *L_m_*	8.6 μH
Snubber Inductor *L_snu_*	4.3 μH
Snubber Capacitor *C_snu_*	150 nF
Input Capacitor *C_in_*	470 μF
Output Capacitor *C_out_*_2_	2700 μF
Power Switches *SW*_1_	IRFP4310
Snubber Diodes *D*_1_, *D*_2_	FCQ30A03L
Rectifier Diode *D*_3_	FCQ03A04
PWM IC	UC3843
Photocoupler	TLP250

**Table 3:** Comparisons of Performance.

Balance Method	Balance Efficiency	Balance Speed	Balance Control	Modular Design
Bleed Resister	Poor	Good	Excellent	Yes
Analog Shunting	Poor	Good	Excellent	Yes
Switched Capacitor	Excellent	Good	Excellent	Yes
Multi-Winding Transformer	Moderate	Excellent	Good	No
Switching Transformer	Good	Moderate	Good	No
Proposed HECT	Good	Good	Moderate	Yes

## Abbreviation Table with Units

**Parameter**	**Unit**
Battery voltage *V_Bn_*	Volt
Switching frequency *f*	Hertz
Output power *P_o_*	Watt
Snubber capacitor *C_snu_*	Farad
Snubber inductor *L_snu_*	Henry
Leakage inductor current *i_Llk_*	Ampere
Input voltage *V_i_*	Volt
Output voltage *V_o_*	Volt
Magnetizing Inductor *L_m_*	Henry
Input Capacitor *C_in_*	Farad
Output Capacitor *C*_*out*2_	Farad
